# Algal pigments: Therapeutic potential and food applications

**DOI:** 10.1002/fsn3.4370

**Published:** 2024-07-29

**Authors:** Ayesha Saddiqa, Zargham Faisal, Noor Akram, Muhammad Afzaal, Farhan Saeed, Aftab Ahmed, Abeer Almudaihim, Muhammad Touqeer, Faiyaz Ahmed, Aasma Asghar, Mubarra Saeed, Gebremichael Gebremedhin Hailu

**Affiliations:** ^1^ Department of Food Science Government College University Faisalabad Faisalabad Pakistan; ^2^ Department of Human Nutrition and Dietetics Iqra University Karachi Pakistan; ^3^ Food Safety & Biotechnology Lab, Department of Food Science Government College University Faisalabad Faisalabad Pakistan; ^4^ Department of Nutritional Sciences Government College University Faisalabad Faisalabad Pakistan; ^5^ Department of Clinical Nutrition King Saud Bin Abdulaziz University for Health Sciences Riyadh Saudi Arabia; ^6^ Department of Basic Health Sciences, College of Applied Medical Sciences Qassim University Buraydah Saudi Arabia; ^7^ Department of Food and Nutrition Government College University Faisalabad Faisalabad Pakistan; ^8^ Food Technology and Process Engineering Oda Bultum University Chiro Ethiopia

**Keywords:** algal pigments, biological properties, food applications, nutrition, therapeutic

## Abstract

Algae‐derived natural compounds have shown significant potential in treating various health conditions, including cancer, obesity, diabetes, and inflammation. Recent advancements in nanotechnology have enabled the development of precise drug delivery systems and diagnostic tools utilizing these compounds. Central to this innovation are the vibrant pigments found in algae chlorophylls, carotenoids, and phycobiliproteins which not only impart color but also possess notable nutritional, medicinal, and antioxidant properties. These pigments are extensively used in supplements and the food industry for their health benefits. Emerging research highlights the role of algal pigments in promoting gut health by modulating gut microbiota. This review comprehensively examines the therapeutic benefits of algae, recent progress in algal‐derived nanoparticle technology, and the synergistic effects of algae and their pigments on gut health. Novel insights and recent data underscore the transformative potential of algal compounds in modern medicine and nutrition.

## Graphical abstract

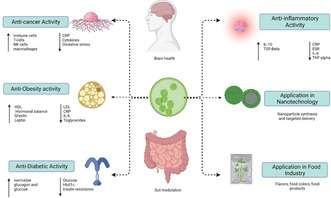

## 1 INTRODUCTION

Algae are a wide‐ranging collection of photosynthetic organisms that live in different aquatic environments, spanning from freshwater to marine ecosystems (Priya et al., 2022). Algae cultivation is highly dependent on certain environmental factors such as temperature, light intensity, pH levels, nutrition availability, and carbon dioxide content. The most favorable circumstances for algae growth are usually found within a temperature range of 20–30°C; however, some species can flourish in colder or hotter environments. Light is essential for photosynthesis, and different species of algae have distinct light needs for their growth and generation of pigments. Moreover, pH levels ranging from 7.0 to 9.0 are generally favorable for the growth of algae; however, certain species may have particular pH requirements (Nouri et al., 2021). The presence of nutrients, specifically nitrogen and phosphorus, is crucial for the growth of algae. The proportions of these nutrients affect the amount of biomass produced and the chemical makeup of the algae. Moreover, the addition of carbon dioxide can significantly increase the growth rates of algae, particularly in enclosed photobioreactors specifically intended for large‐scale culture (Le Gouic et al., 2021).

Algae have been utilized for diverse applications since ancient times, including as a source of food fertilizer, medication, and pigment. Algae have garnered significant interest in recent times due to their potential as a viable source of biofuels, bioproducts, and nanomaterials (Ahmad et al., 2022). Algae, in addition to their primary ecological features, have become a valuable source of bioactive compounds that possess significant medicinal promise (Babich et al., 2022). Algal bioactive compounds, obtained from different species, have demonstrated encouraging outcomes in preclinical and clinical investigations, presenting new opportunities for therapeutic exploration and advancement. Algae have a broad range of therapeutic potential, including their ability to serve as antioxidants, fight against cancer, combat obesity, treat diabetes, and reduce inflammation. The scientific and pharmaceutical professions have shown significant interest in these features, which offer an exciting possibility to create innovative therapeutic substances (Narayanan et al., 2022).

The complex interaction between algae and nanoparticles (NPs) has become a cutting‐edge field of study. The distinct physical and chemical characteristics of NPs, when combined with the bioactive chemicals obtained from algae, collectively amplify their therapeutic effectiveness (Sampath et al., 2022). The combination of nanotechnology and algal‐based medicines has significant potential for the development of precise drug delivery systems, diagnostic instruments, and imaging agents. This has the potential to greatly transform the field of modern medicine. The discussion of algal bioactivity revolves around the vivid pigments that give these organisms their distinct colors (Osório et al., 2020). The pigments, such as chlorophylls, carotenoids, and phycobiliproteins, have both photosynthetic and significant nutritional and medicinal properties. Researchers have been fascinated by the wide range of colors produced by algal pigments, leading to a significant amount of knowledge about their various uses. Algal pigments have been utilized in several practical applications, ranging from supplementing the diet with nutrients while also serving as an antioxidant and natural dye in the cosmetics and food industries (Imchen & Singh, 2022).

There exists a strong and convincing connection between algae, the pigments found in algae, and the health of the gastrointestinal system (Zhou et al., 2022). Recent investigations have revealed their ability to regulate the gut microbiota, an emerging field of study with significant implications for digestive health and nutrition. The complex interaction between chemicals generated from algae and the gut flora signifies the beginning of a new age in tailored nutrition and medicinal interventions. This review paper provides a thorough discussion of the therapeutic possibilities, recent progress in the production and use of NPs derived from algae, and the influence of algae and algal pigments on gut microbiota and gut health. It intends to enhance the existing knowledge by emphasizing the recent progress in algae bioactivity research and highlighting their potential as innovative therapeutic agents. Furthermore, it aims to clearly outline the distinct contributions of this research in relation to previously published material, thereby offering a thorough comprehension of the shifting landscape of algal‐based medicine.

## 2 THERAPEUTIC POTENTIAL OF ALGAE

**FIGURE 1:**
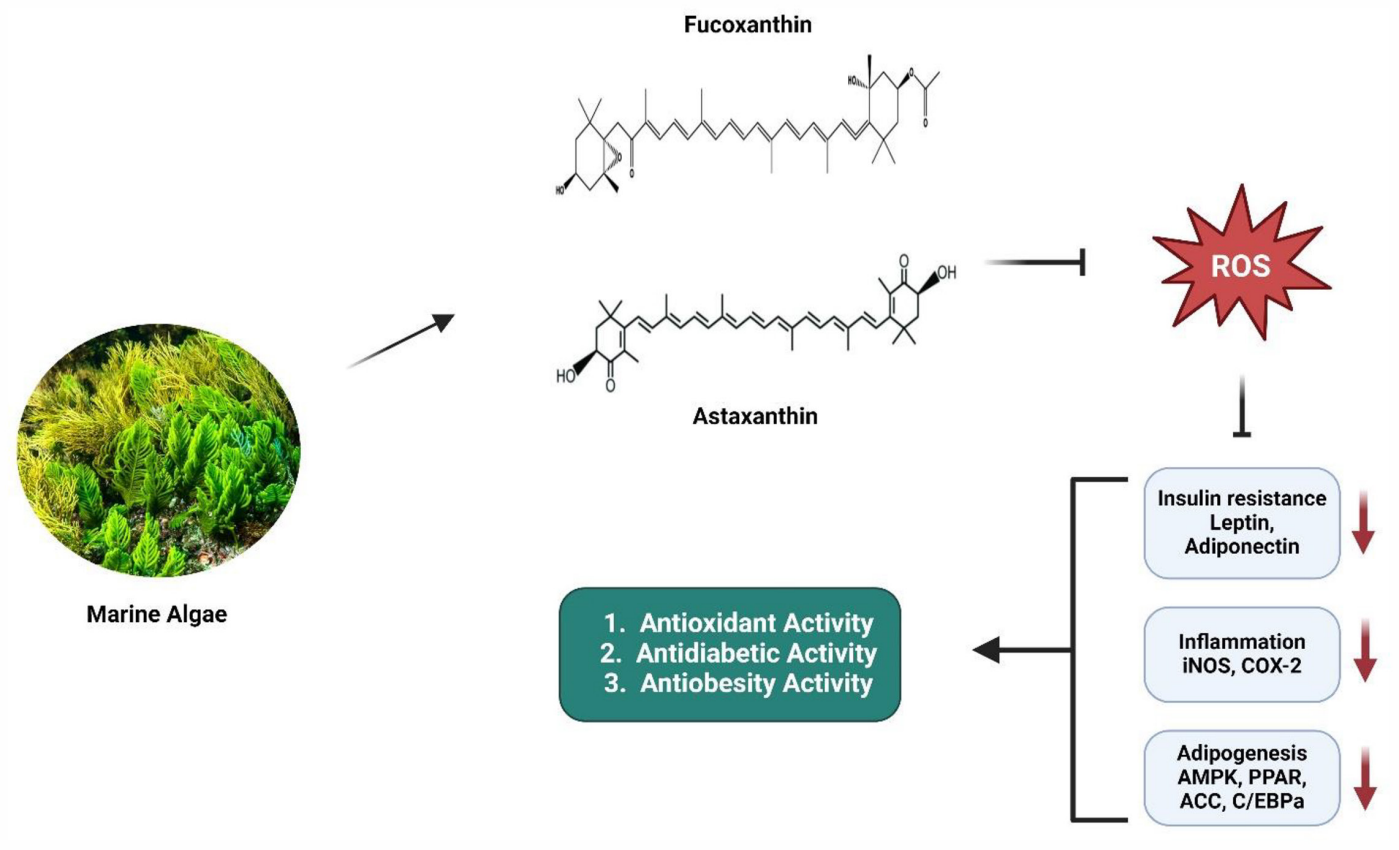
Therapeutic potential of algae.

Algae, a seemingly modest element of nature, possesses significant potential in the field of medicine and health (Ślusarczyk et al., 2021). The varied bioactive compounds it contains have been discovered to possess a wide array of qualities that effectively promote health. Algae has a wide range of therapeutic benefits, including functioning as powerful antioxidants, treating inflammation, and contributing to weight management. This section thoroughly examines these potential possibilities, investigating the extensive and diverse medicinal capabilities of algae. Moreover, therapeutic potential is shown in Figure 1.

### 2.1 Antioxidant activity

Antioxidants are essential for safeguarding the human body against harm induced by reactive oxygen species (ROS). ROS are very reactive substances that can induce oxidative stress, resulting in the impairment of macromolecules such as membrane lipids, proteins, and DNA. The occurrence of oxidative damage has been linked to various health illnesses such as cancer, diabetes, age‐related ailments, and neurological diseases (Luo et al., 2020). Algae, specifically brown and red types, are recognized for their powerful antioxidant properties. The presence of bioactive compounds, including phenolic compounds and carbohydrates, is primarily responsible for these activities. These compounds have demonstrated a strong positive association with antioxidant activities. Furthermore, the antioxidant characteristics of algae make it a highly promising source for the development of functional foods and natural goods (Harb et al., 2021).

Seaweeds are a collection of multicellular algae found in the ocean. The extracts from Seaweed *Chaetomorpha* sp. have attracted interest due to their antioxidant phytochemical contents, which are believed to play a role in preventing human diseases (El‐Beltagi et al., 2022). Haq et al. investigated *Cladophoraceae*, particularly the species *Chaetomorpha* sp. for antioxidant potential. The antioxidant tests revealed that the ethanol extracts of *Chaetomorpha* contained 189.14 ± 0.99 mg QE/g of flavonoids, 21.92 ± 0.43 mg GAE/g of phenolics, and 21.81 ± 0.04 mg GAE/g of tannins. The results were obtained through the examination of the flavonoid composition. The ethanol extract exhibited greater antioxidant activity, as determined by the DPPH radical scavenging experiment, with an IC_50_ value of 9.41 ± 0.54 mg/mL. The study revealed that the investigated species of *Chaetomorpha* possess very effective compounds, making it a promising option for utilization as an antioxidant in the pharmaceutical industry (Haq et al., 2019).

Liu et al. conducted a study using SAMP8 mice to examine the antiaging and antioxidant properties of oligosaccharides obtained from the green algae *Ulva lactuca* (ULO) and *Enteromorpha prolifera* (EPO). Furthermore, they examined the mechanism that supports these characteristics. The oligosaccharides led to a rise in the levels of glutathione, superoxide dismutase, catalase, and telomerase, as well as an overall enhancement in antioxidant capability. In addition, they successfully reduced the concentrations of malondialdehyde and advanced glycation end products. The downregulation of the p53 and FOXO1 genes, along with the upregulation of the Sirt1 gene, suggests that ULO and EPO may have beneficial effects in halting the aging process in SAMP8 mice (Liu et al., 2019). Dell'Anno et al. assessed the antioxidant capacity and antimicrobial properties of the algae species *Ascophyllum nodosum* and *Schizochytrium* spp. against *Escherichia coli* O138. Both *A. nodosum* and *Schizochytrium* spp. had significant antioxidant capacity (*p* < .05). The 1:1 mixture had antioxidant activity, indicating a synergistic impact (*p* < .05). The many characteristics of algae species that can influence the growth of O138 *E. coli*, along with their antioxidant capability, make them a potentially beneficial feed supplement for enhancing gut health (Dell'Anno et al., 2020).

Harb et al. examined the antioxidant activity and chemical composition of methanolic and aqueous extracts from 15 marine algae on the Brazilian Coast. The extracts from brown macroalgae exhibited the highest levels of antioxidant activities, followed by the extracts of red algae. The lowest levels of activities were observed in the green beach‐cast algae. There was a direct relationship between the levels of phenolic compounds and carbohydrates and the antioxidant activity of the extracts that were examined. This study proposes that these algae have the potential to be used as sources for extracting chemicals with antioxidant qualities, particularly phenolic compounds, and sulfated carbohydrates (Harb et al., 2021).

Red seaweeds contain a diverse range of chemicals that possess multiple biological qualities, including antibacterial, antioxidant, antifouling, antiproliferative, and anticancer actions. Hmani et al. conducted research to assess the antibacterial and antioxidative activities of twelve red macroalgae specimens taken from the Tunisian seashore. *Gracilaria gracilis* had the highest amount of total phenolic compounds, measuring 19.2 ± 1.88 mg GAE/g dried biomass. On the other hand, *Laurencia obtusa* had the highest tannin content, measuring 18.95 ± 0.84 mg ECat/g DB. Conversely, *Sphaerococcus cornopifolius* displayed the greatest concentration of flavonoids (7.17 ± 0 mg ECat/g DB). The six distinct species exhibited remarkable DPPH radical scavenging activities and overall antioxidant capacities (Hmani et al., 2021).

A likewise study was conducted to assess the antioxidant activity of four different types of algae found in the soil, including one type that grows on the soil (*Vaucheria geminata*) and three types that were separated from the soil (*Pleurochloris pyrenoidosa*, *Botrydiopsis eriensis*, and *Scenedesmus obliquus*). The phosphomolybdenum assay measured the total antioxidant activity, which varied from 6.66 to 36.33 mg of ascorbic acid per gram of dry weight. Additionally, the DPPH radical inhibition reached a maximum of 97.37%. *B. eriensis* and *S. obliquus* had the most elevated concentrations of phenols, flavonoids, and chlorophyll a, with *P. pyrenoidosa* and *V. geminata* following similarly. Notably, *B. eriensis* displayed the highest content of carotenoids (El‐Tablawy et al., 2020).

### 2.2 Anticancer activity

Algae, due to their abundant bioactive chemicals, have demonstrated promise in restraining the growth and multiplication of cancer cells. This field of study presents novel opportunities for the development of natural, efficacious, and less detrimental therapies for cancer.

Colorectal cancer (CRC) is a prevalent form of cancer and a significant contributor to global cancer mortality. Zbakh et al. conducted a study to examine the anticancer properties of eight algal meroterpenoids (AMTs) obtained from the brown seaweed *Cystoseira usneoides*. The researchers also explored the methods by which these AMTs exert their effects on HT‐29, a human colon cancer cell line known for its strong metastatic potential. All the meroterpenoids that were examined showed inhibitory effects on the proliferation of HT‐29 malignant cells while exhibiting lower toxicity towards noncancerous colon cells. Exposure of HT‐29 cells to the AMTs resulted in the halting of the cell cycle specifically in the G2/M phase, and in certain cases, triggered apoptosis. The findings offer an understanding of the mechanisms and roles of the meroterpenoids found in *C. usneoides*. Evidence has demonstrated that these compounds possess an anticancer property against HT‐29 colon cancer cells, since they are capable of inducing cell cycle arrest and apoptosis (Zbakh, Zubía, De Los Reyes, et al., 2020; Zbakh, Zubía, Reyes, et al., 2020).

*Turbinaria ornata*, a marine macroalgae, exhibits a brown hue and encompasses a diverse range of bioactive constituents that elicit various biological effects. Bharath and colleagues conducted a study using HT‐29 human colon cancer cells to explore the suppressive effect of hexadecanoic acid (HA) generated from *T. ornata*. The antioxidant properties of HA were assessed using the DPPH and Superoxide radical scavenging assays. The IC_50_ values for the DPPH and superoxide radical were determined to be 70.62 and 71.41 μg/mL, respectively. The in vitro anticancer activity of HA demonstrates its potential as a cell growth inhibitor. The IC_50_ value of 36.04 μg/mL indicates its promise in this regard. The investigation of the cell cycle revealed that cell progression was arrested during the G0/G1 phase. This study conducted research into the potential of HA as an anticancer agent (Bharath et al., 2021).

Parthasarathy et al. conducted a study to evaluate the potential of the marine algal endophyte, *Penicillium chrysogenum*, in terms of its ability to combat cancer and bacterial infections. An analysis was conducted on the various organic solvent extracts of endophytic fungi cultivated on different growth mediums to determine their anticancer and antibacterial properties. The most powerful inhibitory action against the MCF‐7 human breast cancer cell line was demonstrated by the ethyl acetate (EA) extract of the culture filtrate, which was grown in potato dextrose broth (PDB) for 21 days (Parthasarathy et al., 2020). A further research investigation was conducted to examine the anticancer and antioxidant properties of the brown algae *Dictyota dichotoma* found along the Western seacoast of Yemen. The evaluation of antioxidant activity was conducted by the utilization of the DPPH radical scavenging test. The extracts of *D. dichotoma* demonstrated a notable cytotoxic effect on the seven tumor cell lines, with the level of toxicity increasing in proportion to the dosage. However, the extracts exhibited a higher degree of selectivity towards MCF‐7 and PC‐3 cells. Out of all the fractions, the chloroform fraction of *D. dichotoma* exhibited the greatest cytotoxic activity and was particularly effective in MCF‐7, PC3, and CACO cells. The petroleum ether fraction exhibited notable efficacy, particularly against MCF‐7 and PC‐3 cell lines, whereas the ethyl acetate fraction had stronger activity against hepatocellular carcinoma (Hip) 2 and CACO cell lines (El‐Shaibany et al., 2020).

An additional investigation assessed the anti‐inflammatory and anticancer characteristics of eight meroterpenoids that were extracted from the brown seaweed *Cystoseira usneoides*. The effects of algal meroterpenoids (AMTs) 1–8 on the production of pro‐inflammatory cytokines tumor necrosis factor (TNF‐α), interleukin‐6 (IL‐6), and interleukin‐1β (IL‐1β), as well as the expression of cyclooxygenase‐2 (COX‐2) and inducible nitric oxide synthase (iNOS), were tested in LPS‐stimulated THP‐1 human macrophages. The cytotoxicity assays were used to evaluate the anticancer effects against human lung adenocarcinoma A549 cells and normal lung fibroblastic MRC‐5 cells. The administration of AMTs 1–8 resulted in a significant decrease in the production of TNF‐α, IL‐6, and IL‐1, and inhibited the expression of COX‐2 and iNOS in cells stimulated with LPS (*p* < .05). The AMTs 1–8 exhibited greater cytotoxicity against A549 cancer cells compared to MRC‐5 normal lung cells. Cell cycle analysis revealed that the majority of the AMTs induced cell arrest in A549 cells specifically at the G2/M and S phases. The results indicate that the AMTs generated by *C. usneoides* could be beneficial in treating inflammatory disorders and lung cancer (Zbakh, Zubía, De Los Reyes, et al., 2020; Zbakh, Zubía, Reyes, et al., 2020).

### 2.3 Antiobesity activity

Obesity has emerged as a global pandemic, with its occurrence steadily rising each year. The absence of physical activity and bad eating habits, driven by modernity, are strongly interconnected. Obesity significantly contributes to the development of metabolic syndrome, which is characterized by diabetes, hypertension, sleep apnea, and cardiovascular diseases (CVDs). Polysaccharides derived from marine algae demonstrate advantageous biological properties. Through the use of in vitro testing, a research study was conducted to evaluate the impact that Codium fragile extract (CFE) has on antiobesity properties. During the process of 3T3‐L1 preadipocytes developing into adipocytes, CFE inhibited the differentiation of adipocytes by increasing the expression of differentiation‐related factors. Hence, it has been proposed that CFE can be employed to achieve an antiobesity impact on human health (Oh et al., 2022).

The objective of this study was to analyze the phytochemical makeup of the ethanol extract of *Nostoc commune* (NEE), a terrestrial alga that is edible, and evaluate its potential in mitigating obesity. This study investigates how a calorically dense meal affects the accumulation of lipids in 3T3‐L1 preadipocytes. Furthermore, the antiobesity effects and mechanism of NEE are demonstrated using a Wistar rat model. The results showed that the NEE had phytochemical components, such as total terpenoids, total flavonoids, and total polyphenols. In 3T3‐L1 preadipocytes, NEE was shown to impede cell proliferation and decrease fat accumulation (by 26.9%). Furthermore, NEE caused a remarkable reduction of 13.5% in body weight, 13.3% in fat tissue weight, 19.4% in blood‐free fatty acid (FFA) levels, 14.2% in triglyceride (TG) levels, 11.8% in total cholesterol (TC), and 16.4% in low‐density lipoprotein cholesterol (LDL‐C) in rats. Adipocyte and hepatic lipid droplet size reduction was shown by histopathological investigation as an effect of NEE. Epididymal adipose tissue‐related genes for lipid lysis (ATGL, HSL) and adipogenesis (PPAR‐, SREBP‐1c) were downregulated by the NEE. The mRNA expression of genes related to β‐oxidation (AMPK, CPT‐1, and PPAR‐γ) in the liver was also enhanced by the NEE. As a functional food that could help fight obesity, NEE shows potential, according to this study (Tsai et al., 2022).

One of the most common bioactive substances in *Ishige okamurae* is diphlorethohydroxycarmalol (DPHC). Ding et al. studied the effects of DPHC on C57BL/6J mice that have gained weight due to a high‐fat diet (HFD). Evidence from this study shows that DPHC, when taken orally for 6 weeks at doses of 25 and 50 mg/kg/day, significantly reduced fat buildup and weight gain due to a high‐fat diet. Serum DPHC levels in HFD mice were found to increase HDL cholesterol while simultaneously decreasing triglyceride, LDL cholesterol, leptin, and aspartate transaminase levels. Furthermore, DPHC dramatically diminished adipocyte size and lowered expression levels of adipogenesis‐related proteins and lipid synthesis‐enzymes (Ding et al., 2019).

In a different study, researchers looked at how the red algae *Gelidium amansii* hot‐water extract (GHE), which is abundant in polysaccharides, could help hamsters overcome their obesity when given HFD. To induce obesity in hamsters, they were fed HFD for 5 weeks. Adding GHE successfully offset the increased body, liver, and adipose tissue masses in the HFD group. GHE reduced lipoprotein lipase activity in adipose tissues and increased lipolysis rate. The plasma and liver triglyceride and total cholesterol levels of obese hamsters who were fed a diet with GHE were shown to be lower (Yang et al., 2019).

Fucoxanthin (FX), a carotenoid derived from marine macroalgae and microalgae, possesses numerous advantageous effects on human health. Koo et al. examined the impact of a standardized FX powder, derived from the microalga *Phaeodactylum tricornutum*, in reducing obesity. This powder, known as *Phaeodactylum* extract (PE), was developed as a functional food for commercial use. Both PE and FX reduced the amount of lipids inside adipocytes in a dose‐dependent manner, without causing any harm to the cells. Supplementing obese mice fed with an HFD with PE for 6 weeks resulted in reductions in body weight, organ weight, and adipocyte size. The study of blood parameters revealed that the groups treated with PE exhibited a reduction in lipid metabolism dysfunction and liver damage caused by HFD. The data suggest that PE has antiobesity effects (Koo et al., 2019).

### 2.4 Antidiabetic activity

Diabetes is a combination of metabolic disorders that impact the body's utilization of blood glucose, a vital energy source for cells and the brain. The etiology of diabetes differs depending on the type, nevertheless, it can result in severe complications such as cardiovascular disease, renal failure, and neuropathy (Nabrdalik et al., 2023). Figure 1 illustrates the antidiabetic, antioxidant, and antiobesity activity of astaxanthin and FX derived from marine algae. Six different brown algae were tested in an experimental setting from 2017 to 2019 for their ability to suppress α‐glucosidase activity. *Colpomenia sinuosa*, *Sargassum acinaciforme*, *Iyengaria stellata*, *Sirophysalis trinodis*, and two different accessions of *Polycladia myrica* were all examined in their methanol (MeOH) and 80% MeOH extracts. *Colpomenia sinuosa* and *Iyengaria stellata* 80% MeOH extracts, as well as *Colpomenia sinuosa* MeOH extracts, showed a more potent inhibitory action on α‐glucosidase than acarbose. Postmeal blood glucose levels in diabetic rats were lower when treated with 80% *Sirophysalis trinodis* extracts compared to the control group. In most of the algae extracts, FX was identified as the primary antidiabetic component (Moheimanian et al., 2022).

Shan et al. demonstrated that *F. vesiculosus* fucoidan has the capacity to block α‐glucosidase in a laboratory setting and effectively reduce fasting blood glucose and HbA1c levels in diabetic mice (Shan et al., 2016). A study was conducted to investigate the impact of fucoidan derived from *U. pinnatifida* on the accumulation of lipids, the breakdown of lipids, and the uptake of glucose in 3T3‐L1 cells. The study demonstrated that the sulfated polysaccharide effectively decreased the buildup of lipids and the activity of glycerol‐3‐phosphate dehydrogenase in a manner that depended on the dosage (*p* < .01). The administration of fucoidan also resulted in the inhibition of PPARγ expression, a prominent transcription factor involved in the process of adipocyte development. The results indicate that fucoidan may possess antidiabetic properties via enhancing insulin‐mediated glucose absorption and suppressing basal lipolysis in adipocytes, without promoting adipogenesis (Sim et al., 2019).

Xu et al. discovered that phlorotannins derived from the brown alga *Ecklonia kurome* have a beneficial effect on diabetes in KK‐Ay mice, which are used as an animal model for human type 2 diabetes mellitus (T2DM). The carbohydrate‐hydrolyzing enzymes, especially α‐glucosidase, were inhibited by algae, while α‐amylase was significantly suppressed. As shown in in vivo experiments, this led to a decrease in postprandial blood glucose levels. When compared to a control group, those who consumed polyphenols from *E. kurome* had better glucose tolerance and lower fasting blood glucose, insulin, fructosamine, and glycoalbumin levels (Xu et al., 2017).

### 2.5 Anti‐inflammatory activity

Algae, which comprise marine algae, microalgae, and cyanobacteria, are abundant reservoirs of bioactive substances, including peptides, fatty acids, and polysaccharides, which possess anti‐inflammatory activities. Algae, due to their diverse methods of action, both inside and outside cells, show potential as a valuable source of anti‐inflammatory substances for treating associated disorders (Tabarzad et al., 2020). An investigation was carried out to create and assess the anti‐inflammatory and cytotoxic properties of the Methanolic extract of *Chondrus crispus* (MEC), a red algal species. The MEC's ability to significantly reduce bovine serum albumin denaturation suggests it may have anti‐inflammatory properties. Furthermore, 81.9% of HepG2 cells and 71.8% of adenocarcinoma human alveolar (A549) cells perished from cytotoxic effects after being treated with MEC. In contrast, cells treated with the standard medication sorafenib had a cell death rate of 69% (Alkhalaf, 2021).

The effects on structural characteristics and anti‐inflammatory activities of algal sulfated polysaccharides produced by *Gracilaria lemaneiformis* were the subject of a distinct study. In vitro, GLP's anti‐inflammatory effects were strongest after 5 min of administration. The result was a 60.49% decrease in nitric oxide (NO) production, a 62.81% decrease in tumor necrosis factor‐α production, and a 36.29% decrease in IL‐6 production in IEC‐6 cells compared to the control group (Gong et al., 2021). To understand the molecular mechanism underlying LPS‐induced acute liver and kidney injury in mice with endotoxemia, researchers examined the effects of *Galaxaura oblongata* (*G. oblongata*), red algae, on inflammation, apoptosis, and oxidative stress levels. *G*. *oblongata* extract's antioxidant capacity and antibacterial activity could explain why it reduced liver apoptosis by blocking the protein tyrosine kinase signaling pathway and decreased serum cytokines like NF‐κB and myeloperoxidase in rats when pretreated with the extract (Nabil‐Adam & Shreadah, 2021).

*Prasiola japonica* exhibits multiple biological actions. Rahmawati et al. conducted a study to examine the possible anti‐inflammatory effects of *P. japonica* ethanol extract (Pj‐EE) and its four solvent fractions: hexane (Pj‐EE‐HF), chloroform (Pj‐EE‐CF), butanol (Pj‐EE‐BF), and water (Pj‐EE‐WF). The investigation involved both in vitro and in vivo studies. The most significant suppression of NO generation was reported in Pj‐EE‐CF during in vitro experiments. Pj‐EE‐CF also decreased the expression of genes and the generation of cytokines in tissue lysates of paw edema produced by carrageenan (Rahmawati et al., 2021).

## 3 ALGAE AND NANOPARTICLES

Algae, a varied array of photosynthetic organisms, have demonstrated significant promise in the world of nanotechnology. Due to their distinct metabolic processes, they can synthesize a diverse range of bioactive molecules. These compounds can then be utilized to create NPs that have both medicinal and industrial uses. Recent research has emphasized the involvement of algae in the production of NPs through biosynthesis, offering an environmentally friendly and sustainable method for nanotechnology (Chan et al., 2022).

In their description of the process, Araya‐Castro et al. used specific protein fractions isolated from a water‐based extract of the brown alga *Macrocystis pyrifera* to create copper oxide NPs. Size exclusion chromatography was used to separate these protein fractions. The samples under investigation had metallic cores with diameters ranging from 2 to 50 nm, as shown by transmission electron microscopy scans. The protein samples were also uniformly nanostructured on a spherical basis. The functional groups of proteins are essential for decreasing and stabilizing NPs, as shown by FTIR studies. The wide range of possible uses for NPs is attributed to their exceptionally stable nature, as seen by their highly negative average zeta potential values (Araya‐Castro et al., 2020).

Palladium NPs (Pd‐NPs) were produced utilizing an environmentally friendly technique by an extract derived from a brown alga called *Padina boryana* (PB‐extract). The synthesized Pd‐NPs were then assessed for their antibacterial, antibiofilm, and anticancer properties. The data show that the Pd‐NPs are crystalline and have an average diameter of 8.7 nm. The zeta potential is −28.7 ± 1.6 mV, and the hydrodynamic size is 48 nm. Pd‐NPs showed strong antibacterial and antibiofilm capabilities against several strains, with an inhibitory minimum dose varying between 62.5 and 125 μg/mL. Investigations into cell viability showed that the proliferation of breast cancer MCF‐7 cells was inhibited, with the effect being concentration‐dependent. The messenger RNA expression of genes that indicate cell death was also upregulated by Pd‐NPs. To effectively treat breast cancer cells and multidrug‐resistant bacteria in the clinic, this study suggested using PB‐extract capped Pd‐NPs (Sonbol et al., 2021).

The production of nanoliposomes containing algal extract was optimized using the central composite response surface design. Nanoliposomes were prepared using varying amounts of phenolic chemicals from algal extract and lecithin. The loaded nanoliposomes exhibited a particle size ranging from 86.6 to 118.7 nm and a zeta potential ranging from −37.3 to −50.7 mV. Evidence has shown that the developed nanoliposomes possess excellent durability under storage circumstances and effectively regulate the release of phenolic chemicals across various pH levels. The antioxidant activity of the algal extract has been preserved at a satisfactory level during the encapsulating process. Therefore, nanoliposomes containing algal extract can serve as a natural antioxidant in lipid‐based food products (Savaghebi et al., 2020).

To create ZnO NPs, Thirumoorthy et al. used zinc acetate reduction. They used a green synthesis approach that included the use of *Tetraselmis indica* algal extract as a precursor. Experiments using an X‐ray diffractometer (XRD) verified that the nanoparticle had a crystalline spherical structure, and the average size of the ZnONPs was found to be about 27 nm. Using FTIR, the researchers were able to separate the compounds into multiple functional groups with their own unique peak locations. Images captured by scanning electron microscopy (SEM) revealed the 20–40 nm sized zinc oxide NPs. The hemolysis assay conducted using horse blood demonstrated that the NPs had no toxicity toward the erythrocytes. ZnO NPs exhibited a significant reduction property in high concentrations (100 mg/mL), indicating a strong free radical scavenging activity that is directly proportional. Hence, the observed findings not only suggest the potential utilization of ZnO NPs but also present algae as an alternative and cost‐effective source for synthesizing ZnO NPs through environmentally friendly methods. These findings demonstrate the potential applications of ZnO NPs in various fields such as biomedicine, textiles, cosmetics, and food packaging (Thirumoorthy et al., 2021).

Another research looked at the effectiveness of silver nanoparticles made from the marine algae *Chaetomorpha linum* as an anticancer agent against the HCT‐116 human colorectal cancer cells in a controlled laboratory environment. The cytotoxic effect of biosynthesized silver NPs on HCT‐116 was determined to be dose‐dependent using the MTT assay. Research on cell death found that antiapoptotic proteins such as Bcl‐2 and Bcl‐xl were downregulated, while apoptotic caspase 3, caspase 9, BH3‐interacting domain death agonist (Bid), and Bax were upregulated. As an anticancer agent, the biologically synthesized C‐AgNPs were highly effective in causing cell death in HCT‐116 colon cells (Acharya et al., 2021).

Using extracts from different marine algae species as reducing and capping agents, biogenic silver nanoparticles (AgNPs) were created. These algae species include green alga *Ulva rigida*, brown alga *Cystoseira myrica*, and red alga *Gracilaria foliifera*. A variety of cell lines, including those from normal skin (HFb‐4), breast cancer (MCF‐7), and Artemia salina nauplii, were used to assess the cytotoxicity and anticancer characteristics of the biosynthesized AgNPs. It was shown that the mortality rate was proportional to the concentration of AgNP. The antifungal action of AgNPs against dermatophyte pathogenic molds was strong, and the antibacterial action against microorganisms that cause food poisoning was limited. Researchers found that extracts from *U. rigida* can be used as reducing agents in a green way to make AgNPs. These AgNPs have anticancer properties against the MCF‐7 cell line and are considered an effective alternative treatment for dermatophyte‐induced skin infections (Algotiml et al., 2022).

To get the most carbs out of *Pterocladia capillacea*, Aboeita et al. optimized ultrasound‐assisted extraction to get the best yield feasible. The extract served as a reducing and capping agent in the green synthesis of copper NPs. The primary objective was the production of CuO NPs, which typically have a size of 62 nm. The extract and the CuO NPs mediated by algae showed distinct peaks in the polysaccharides FTIR spectra. Nedaplatin was loaded onto the CuO NPs after their production. After 120 h, the nedaplatin release profiles peaked, indicating a sustained release. Cell lines from hepatocellular carcinoma, breast cancer, and ovarian cancer showed that the formulation was more cytotoxic than nedaplatin. Adding nedaplatin to CuO NPs, which were made from red algal extract, makes them much more effective against cancer (Aboeita et al., 2022).

Algae's employment in the production of NPs has created new opportunities in the realm of nanotechnology. Algae's wide range of metabolic capacities not only allows for a sustainable and environmentally friendly method of synthesizing NPs but also presents a significant opportunity for uncovering new applications in many disciplines (Khan et al., 2022).

## 4 ALGAL PIGMENTS

Algae, a varied collection of organisms that can perform photosynthesis, display a remarkable spectrum of pigments, which contribute to their vibrant hues and distinct biological adaptations. Chlorophylls, the main pigments accountable for the green tint, have a crucial function in photosynthesis (Taniguchi et al., 2022). Chlorophyll‐*a*, which is the predominant type, exhibits the highest light absorption efficiency in the red and blue wavelengths. Chlorophyll‐*b* supplements its absorption specifically at the green wavelength, augmenting the total process of photosynthesis (Glemser et al., 2022).

Algae possess a range of carotenoids, alongside chlorophylls, which are accountable for the diverse shades of red, orange, and yellow seen in different species. These lipophilic molecules, such as β‐carotene, lutein, and zeaxanthin, function as supplementary pigments, expanding the spectrum of light absorption and safeguarding cells against excessive light energy (Lim et al., 2023). The brownish color of brown algae is attributed to the presence of FX, a pigment that is exclusive to this group. FX, in addition to being a pigment, has significant antioxidant qualities and has attracted attention for its possible health advantages, particularly in metabolic disorders (Miyashita et al., 2020).

Phycobilins, which are unique to red and blue‐green algae, distinguish them based on their pigmentation characteristics. Phycocyanin, a pigment that appears blue, and phycoerythrin, a pigment that appears red, are the basis for their colors. The water‐soluble pigments have a crucial function in absorbing light, which improves their ability to carry out photosynthesis more effectively in deeper water conditions (Simkin et al., 2022).

### 4.1 Nutritional and therapeutic significance

The vibrant pigmentation of algae reflects their high nutritional content, making them a vital addition to both human and animal diets (Demarco et al., 2022). The medicinal potential of algal pigments has become prominent in the pharmaceutical and nutraceutical industries. Chlorophyll, commonly known as “green blood,” possesses structural resemblances to hemoglobin and has attracted attention due to its potential to improve human health. The antioxidant properties of this substance aid in the process of detoxification and provide cellular protection against oxidative stress. Moreover, the abundant presence of magnesium in chlorophyll is crucial for numerous physiological functions (Ye et al., 2019). Carotenoids have significant nutritional significance in addition to their function as pigments. β‐carotene, a precursor of vitamin A, promotes ocular health, enhances immunological function, and maintains skin integrity. β‐carotene functions as an agent that eliminates harmful free radicals, specifically singlet oxygen and peroxyl radicals, thus safeguarding cells and biomolecules from oxidative harm. This action is crucial for safeguarding cellular components such as lipids, proteins, and DNA against oxidative stress, which is linked to numerous disorders such as cancer, cardiovascular diseases, and aging (Bhatt & Patel, 2020). In the body, β‐carotene is transformed into retinol (also known as vitamin A) through enzymatic cleavage by β‐carotene 15,15′‐dioxygenase in the intestinal mucosa. Retinol is essential for eyesight, immunological function, cell proliferation, and differentiation. β‐carotene acts as a precursor to vitamin A, which is crucial for sustaining general health. β‐carotene boosts the function of natural killer cells, lymphocytes, and macrophages, hence enhancing the body's immune response to infections and tumors. It hinders the development of cancer by removing free radicals, causing cancer cells to undergo programmed cell death, and disrupting the growth and spread of cancer cells (Koklesova et al., 2020).

Lutein and zeaxanthin, mostly present in green algae, enhance ocular well‐being and potentially mitigate the likelihood of age‐related macular degeneration (AMD). Lutein is found in high levels in the macula of the eye, where it functions as a natural barrier against blue light and safeguards against damage caused by photooxidation. Lutein preserves the structural integrity of the macular pigment and safeguards against age‐related eye illnesses such as AMD, cataracts, and other conditions by absorbing excessive blue light and neutralizing reactive oxygen species produced by light exposure (Mrowicka et al., 2022). Lutein counteracts the harmful effects of oxidative stress, safeguarding cells, tissues, and organs against damage resulting from oxidative reactions. This, in turn, lowers the likelihood of developing chronic diseases. Lutein aids in preserving vascular health by strengthening the function of the endothelium, increasing the availability of NO, and preventing the development of atherosclerotic plaques. As a result, it reduces the likelihood of heart attacks, strokes, and other cardiovascular incidents. Mounting evidence indicates that consuming lutein is linked to improved cognitive performance, specifically in areas such as memory, processing speed, and executive function. Additionally, it may aid in lowering the likelihood of age‐related cognitive decline and neurodegenerative disorders such as Alzheimer's disease (Maci, 2021).

FX, mostly present in brown algae, demonstrates antiobesity and antidiabetic characteristics. The distinctive way it operates in fat tissue and liver metabolism has stimulated study into its possible medicinal uses (Gille et al., 2019). FX is thought to enhance thermogenesis in both white adipose tissue (WAT) and brown adipose tissue (BAT). Thermogenesis is the process of converting energy into heat, which can raise metabolic rate and aid in fat burning. UCP1 is a protein present in brown adipose tissue that has a vital function in thermogenesis. It achieves this by decoupling oxidative phosphorylation from ATP synthesis, leading to the creation of heat. FX has demonstrated the ability to increase the expression of UCP1, which enhances thermogenesis and energy expenditure (Wu et al., 2014). FX demonstrates anti‐inflammatory effects by inhibiting the synthesis of pro‐inflammatory molecules, including IL‐6, TNF‐α, and COX‐2. FX has demonstrated the ability to trigger apoptosis in many types of cancer cells such as those found in breast, prostate, colon, and lung cancer. FX has the ability to regulate the signaling pathways that are responsible for cell proliferation, survival, and metastasis. These pathways include PI3K/Akt, MAPK/ERK, and Wnt/β‐catenin pathways. FX has been found to suppress hepatic gluconeogenesis, the metabolic pathway in the liver responsible for glucose production, hence potentially aiding in the control of diabetes (Mumu et al., 2022).

Phycocyanin, a blue pigment derived from Spirulina, exhibits anti‐inflammatory, antioxidant, and immunomodulatory properties. The application of this treatment has demonstrated potential in mitigating ailments such as arthritis and inflammatory bowel illness. Phycocyanin hinders the function of pro‐inflammatory enzymes including COX‐2 and lipoxygenase (LOX), thereby diminishing the production of prostaglandins and leukotrienes that contribute to inflammation (Talero et al., 2015). Additionally, it boosts the synthesis of cytokines, such as interferon‐gamma (IFN‐γ) and interleukin‐2 (IL‐2), which are vital for immunological control and protection against infections. Furthermore, the emphasis on phycocyanin's ability to alleviate neurodegenerative illnesses caused by oxidative stress has increased (Agrawal et al., 2021). Astaxanthin, a red pigment present in microalgae and crustaceans, has earned acknowledgment for its powerful antioxidant characteristics. The fact that it can penetrate the blood–brain barrier indicates possible neuroprotective advantages, making it a focal point for research in neurodegenerative disorders. The therapeutic potential of astaxanthin is emphasized by its involvement in promoting skin health, providing cardiovascular protection, and enhancing eye health (Donoso et al., 2021). It scavenges the free radicals produced by UV radiation, suppresses inflammatory reactions in the skin, and stimulates the synthesis of collagen and other components of the extracellular matrix, resulting in enhanced skin flexibility and reduced wrinkles. By reducing inflammation in the vascular endothelium, inhibiting LDL cholesterol oxidation, and increasing NO production, it aids in maintaining healthy blood flow and blood pressure (Pereira et al., 2021).

Phycoerythrin, the predominant red pigment found in red algae, has attracted attention due to its potential utility in photodynamic treatment (PDT). Upon light activation, it possesses the capacity to produce reactive oxygen species, specifically targeting cancerous cells. This indicates a potential role in the treatment of cancer, especially in situations where traditional medicines may have limitations (Zang et al., 2020). Phycoerythrin's fluorescence characteristics and ability to interact well with living organisms make it very ideal for a range of imaging and diagnostic methods, as well as for an array of medical treatments (Qiang et al., 2021).

### 4.2 Applications of algal pigments

Algae's vivid pigments are widely used in several sectors. Within the food industry, natural colorants are utilized as substitutes for synthetic dyes. Spirulina and Chlorella, which contain high levels of phycocyanin and chlorophyll, are used in the manufacturing of functional foods and health supplements. In addition to providing color, algal pigments also contribute to enhancing flavor and nutrition, which aligns with the growing desire for natural and health‐promoting ingredients (Pina‐Pérez et al., 2019).

Carotenoids and chlorophylls are natural pigments used in cosmetics to provide color and antioxidant benefits. The inclusion of these compounds in skincare products corresponds to the increasing desire for natural and environmentally friendly components. Furthermore, the beauty sector has shown interest in the possibility of astaxanthin in topical formulations for protecting the skin against damage caused by UV radiation (Eren et al., 2019).

The pharmaceutical sector utilizes the medicinal properties of algal pigments. Phycocyanin, FX, and astaxanthin have been utilized in formulations aimed at addressing different health issues, offering a promising path for future pharmaceutical advancements (Table 1). Their ability to be absorbed and utilized by the body, as well as their diverse impact on various cellular activities, provide a distinct opportunity for advancements in the field of pharmaceuticals (Vieira et al., 2020).

**TABLE 1:** Functions and practical applications of algal pigments.

Algal pigment	Source	Applications and functions	Bioavailability	Reference
Chlorophyll	*Chlorella vulgaris*, *Scenedesmus obliquus*	Natural green colorant in food and beverages, antioxidant, wound healing, deodorant, and skincare products	Poorly absorbed in intact form, may undergo partial breakdown by gut microbiota to release phytol and chlorophyllin	Aly et al. (2023), Nabi et al. (2023), Zhong et al. (2021)
Astaxanthin	*Haematococcus pluvialis*, *Xanthophyllomyces dendrorhous*	Red pigment used is fish and poultry feed, natural colorant in food, antioxidant, antiaging, and skin‐protecting cosmetics	Lipophilic nature, well absorbed in the gastrointestinal tract and exhibits high bioavailability	Dutta et al. (2023), Kumar et al. (2022), Madhavi et al. (2018)
β‐Carotene	*Dunaliella salina*, *Blakeslea trispora*	Orange pigment is used as a food colorant, antioxidant, provitamin A, cancer prevention, and immune system booster	Converted to Vitamin A through enzymatic cleavage by β‐Carotene 15,15′‐monooxygenase, bioavailability depends upon dietary fat intake, gut health, and variations in enzymatic activity	Anand et al. (2022), Desmarchelier and Borel (2017), Imchen and Singh (2022)
Canthaxanthin	*Chlorella vulgaris*, *Aspergillus carbonarius*	Natural colorant, antioxidant, and tanning agent	Depends upon the formulation and food matrix in which it is consumed	Biskanaki et al. (2023), Duan et al. (2024), Gaur and Bera (2023)
Fucoxanthin	*Phaeodactylum tricornutum*, *Sargassum serratifolium*	Natural colorant, antiobesity, antidiabetic, anticancer	Limited bioavailability due to low solubility and metabolic conversion to more bioactive metabolites i.e., fucoxanthinol	Chini Zittelli et al. (2023), Mumu et al. (2022), Sun et al. (2023)
Zeaxanthin	*Parachlorella* sp., *Navicula clavata*	Antioxidant, eye creams and skincare products, and anticancer	Absorbed in gastrointestinal tract and transported to tissues like retina	Dussably et al. (2022), Patel et al. (2022), Tudor and Pintea (2020)
Lutein	*Muriellopsis* sp., *Tetraselmis suecica*	Natural colorant, antioxidant, eye health	Fat‐soluble pigment, bioavailability enhances when taken with fatty food	Becerra et al. (2020), Kadri et al. (2023), Kim et al. (2023)
Phlorotannins	*Ecklonia cava*, *Sargassum muticum*	Antioxidant, antidiabetic, antiobesity, anticancer	May undergo metabolism of gut microbiota, fiber, and protein in food can affect their release and absorption	Cassani et al. (2020), Phang et al. (2023), Pradhan and Ki (2023)
Phycocyanin	*Spirulina platensis*	Natural blue colorant used in confectionery, anti‐inflammatory, immunomodulatory	Protein pigment in nature, requires proteases to cleave and absorb, smaller peptides and amino acids are more readily absorbed	Ashaolu et al. (2021), Fernandes et al. (2023), Liu et al. (2022)
Sulfated polysaccharides	*Porphyridium cruentum*, *Pterocladia capillacea*	Antioxidant, anticoagulant, antiviral, and moisturizer	Require specific enzymes like sulfatases for their breakdown, larger molecular weight can reduce bioavailability	Ahsan (2019), Figueroa et al. (2022), Nagahawatta et al. (2023)

## 5 ALGAE, ALGAL PIGMENTS, AND GUT HEALTH

The gut microbiome (GMB), an intricate group of bacteria inhabiting our digestive tract, has a vital function in preserving our well‐being (Livovsky et al., 2020). It facilitates the process of digesting food, enhances the body's defense mechanism, and has an impact on our emotional state. Algae, known as the “green gold” of nature, are not merely inhabitants of ponds but rather possess additional characteristics. Algae and algal pigments are becoming recognized as powerful regulators of gut health. They have been discovered to exhibit prebiotic characteristics, which stimulate the proliferation of advantageous intestinal microorganisms. In addition, several algal pigments possess anti‐inflammatory and antioxidant properties, which additionally enhance the overall health of the gut environment (Ávila‐Román et al., 2021).

Mounting research has shown that non‐starch polysaccharides have a substantial impact on the composition, variety, and abundance of the GMB. For instance, the polysaccharides found in Cordyceps sinensis enhanced the diversity of microbial communities and influenced the overall composition of GMB. This was achieved by boosting the presence of beneficial bacteria such as *Lactobacillus*, *Bifidobacterium*, and *Bacteroides*, while reducing the levels of harmful bacteria like *Clostridium* and *Flexispira* (Ying et al., 2020). The polysaccharides from Sargassum pallidum controlled the makeup of gut microbiota by decreasing the ratio of Firmicutes to Bacteroidetes and boosting the prevalence of certain beneficial genres, including *Prevotella*, *Dialister*, *Phascolarctobacterium*, *Ruminococcus*, and *Bacteroides* (Yuan et al., 2020). Another study found that the polysaccharides (JHP) derived from *Coreopsis tinctoria* can undergo partial degradation during digestion in the gastrointestinal tract. However, the indigestible portion of JHP (JHP‐I) can still be further broken down and utilized by GMB. Simultaneously, JHP‐1 facilitates the proliferation of certain advantageous bacteria, including *Bifidobacterium*, *Lactobacillus*, *Megamonas*, and *Megasphaera* species (Wu et al., 2021).

The biological roles of natural algal polysaccharides and carotenoids have been documented, and their influence on the gut microbiota is currently a subject of investigation. Pratap et al. investigated the impact of ulvan, a sulfated polysaccharide, and astaxanthin, a carotenoid, extracted and purified from *Ulva ohnoi* and *Haematococcus pluvialis*, respectively, on the growth of the gut microbiota in mice over time. Noticeable alterations in the bacterial groups *Bacteroidia*, *Bacilli*, *Clostridia*, and *Verrucomicrobia* were found following the administration of ulvan and astaxanthin to the mice. The results emphasize the capacity of ulvan and astaxanthin to regulate several aspects of the mutually beneficial relationship between the host and microbes in the gut. If included in the diet, these substances could have a good impact on treating ailments and conditions related to gut health (Pratap et al., 2022).

Microalgae, including *Phaeodactylum tricornutum* (PT), serve as a renewable and environmentally‐friendly reservoir of valuable nutrients, notably eicosapentaenoic acid (EPA), FX, and chrysolaminarin (Chrl). Stiefvatter et al. produced three distinct diets consisting of microalgae high in either EPA and Fx, Chrl, or an isocaloric control diet (CD). Both diets including microalgae resulted in a rise in the production of specific short‐chain fatty acids (SCFAs) and a drop in the ratio of *Firmicutes* to *Bacteroidota*. Additionally, the diet high in Chrl led to an increase in the presence of *Akkermansia*. This preclinical investigation showed that PT is appropriate for inclusion in the diet of mice, resulting in favorable alterations in the makeup of their microbiota and increased synthesis of SCFAs. These findings indicate potential benefits for gut health (Stiefvatter et al., 2022).

Recent studies have examined carotenoids as adjuvants to treat some chronic and inflammatory disorders that may affect GMB. While the health advantages of carotenoids are widely documented, only a fraction of the carotenoids consumed through diet are absorbed by the intestines and subsequently distributed to the body's tissues. The remaining substances are transported into the colon and undergo metabolism by the bacteria (Bas‐Bellver et al., 2020). There have been reports indicating that carotenoids possess potential prebiotic properties. Carotenoid supplementation can enhance the abundance and variety of the microbial population, resulting in comprehensive alterations in the microbial community. In humans, an increased intake of carotenoids in the diet has been found to effectively suppress the growth of certain types of *Firmicutes* bacteria that are strongly linked to being overweight or obese (Guo et al., 2019).

de Medeiros et al. assessed the possible impact of microalgae biomass (*Chlorella vulgaris*, *Desmodesmus maximus*, *Chlorococcum* sp. cf *hypnosporum*, and *Spirulina platensis*) on human health using an in vitro gastrointestinal digesting model and a human fecal fermentation model. The microalgae biomass demonstrated a notable regulatory impact on the GMB, including an increase in the proportion of *Bifidobacterium* and *Lactobacillus‐Enterococcus*, and a decrease in the presence of undesirable bacteria such as *Eubacterium rectale* group, *Clostridium coccoides*, and *Prevotellaceae* (de Medeiros et al., 2021).

A different study was conducted to analyze the effects of chlorophyllin, which is produced from the green pigment chlorophyll, on the gut microbiota, intestinal mucosal barrier, and liver fibrosis. The findings demonstrated that the ingestion of chlorophyllin orally could reduce inflammation in the intestines and liver, as well as improve liver fibrosis. Crucially, when chlorophyllin was taken orally, it quickly restored the balance of microorganisms in the gut. This was achieved by reducing the presence of the *Firmicutes* group and increasing the presence of the *Bacteroidetes* group (Zheng et al., 2018).

## 6 CONCLUSION

Algae and their pigments provide a large source of bioactive compounds that have substantial medicinal potential. These organisms have a wide range of uses in health and medicine, including their ability to act as antioxidants, fight against cancer, and regulate intestinal health. Future research should focus on improving strains, optimizing extraction methods, and creating effective formulations to enhance therapeutic effectiveness. Likewise, studying the complex relationship between algal pigments and intestinal microbes can provide opportunities for personalized dietary treatments. The combination of algae and NPs offers promising prospects for precise medication delivery and diagnostic progressions. In addition, algal pigments, which have nutritional and medicinal importance, are used in several industry sectors. Future investigations are necessary to fully exploit the capabilities of algae and their components. Comprehending the intricate interplay between algae compounds and the gut flora shows potential for tailored dietary therapies. Furthermore, the incorporation of algae‐based technologies in the fields of nanomedicine and biotechnology presents a promising opportunity for groundbreaking advancements.

## AUTHOR CONTRIBUTIONS

**Ayesha Saddiqa:** Writing – review and editing (equal). **Zargham Faisal:** Writing – original draft (equal); writing – review and editing (equal). **Muhammad Afzaal:** Editing, Supervision. **Farhan Saeed:** Project Administartion. **Noor Akram:** Writing – original draft (equal); writing – review and editing (equal). **Aftab Ahmed:** Investigation (equal); methodology (equal). **Abeer Almudaihim:** Data curation (equal); software (equal). **Muhammad Touqeer:** Visualization (equal). **Faiyaz Ahmed:** Software (equal); writing – review and editing (equal). **Mubarra Saeed:** Visualization (equal). **Aasma Asghar:** Visualization (equal). **Gebremichael Gebremedhin Hailu:** Editing, Writing.

## FUNDING INFORMATION

The authors declare that no funds, grants, or other support were received during the preparation of this manuscript.

## CONFLICT OF INTEREST STATEMENT

The authors declare that they have no known competing financial interests or personal relationships that could have appeared to influence the work reported in this paper.

## Data Availability

Even though adequate data have been given in the form of tables and figures, however, all authors declare that if more data are required then the data will be provided on a request basis.
